# Castrations Statistics

**Published:** 1883-10

**Authors:** W. S. Adams


### Art. XXVII.—CASTRATION STATISTICS.

BY W. S. ADAMS, V.S.

*From the Quarterly Journal of Veterinary Science in India.*

THE register of Castration at the Remount Depot contains a record of over 10,000 cases, and I have thought that some interesting or valuable information might be collected therefrom.

Before the operation, each horse receives a dose of physic, and after the effects of the purgative have passed off, grain is given for three days. The horse is operated upon on the morning of the fourth day. The vascular portion of the cord is divided by the scraping method, a knife with a serrated edge, made for the purpose, is used. On the day of the operation the horse is kept as quiet as possible, and his head is tied up with a rope long enough to permit him to lie down. On the following morning he is let loose in his stall. On the morning of the third day all the coagula are removed from the scrotum, the parts cleansed and dressed. On the evening of the same day a short walk is given. The wounds are cleansed morning and evening and the walk is gradually prolonged. On the day of operation the feed is reduced and an equivalent of bran is given. On the eighth day after the operation full feed is allowed. The horse is discharged off sick-report usually about the twenty-first day.

The chief causes of death from the operation and the percentage of deaths on the total number of operations, 10,305, are as follows:—

| | | | |
|---|---|---|---|
| Peritonitis, | 116 | — | 1.12 |
| Gangrene, | 76 | — | .73 |
| Hœmorrhage, | 22 | — | .21 |
| Tetanus, | 16 | — | .15 |

In considering this mortality it is necessary to remember that the age of the horses operated on is from four to six years; but there are many rising four, and six off.

*Peritonitis.*—I am inclined to the opinion that there is a constitutional tendency to this disease which is aggravated by exposure to inclement weather, or by the depressing effects of extreme heat.

The attack is ushered in by severe rigors accompanied by profuse cold perspiration, and the horse is found pulseless. As soon as these cases occur the patient is rubbed as dry as possible, warmly clothed, ears pulled, legs hand-rubbed and bandaged, and a strong stimulant given. These cases generally occur from the third to the sixth day after operation. In the majority the rigors pass off, but in some they appear to be premonitory of acute peritonitis.

These "cold fits," as they are termed, puzzled me very much at first. I was in doubt whether they were due to faintness from simple exhaustion, or to the disturbance of the system on the formation of pus, but on observation I have concluded that these rigors are premonitory of peritonitis, and that if prompt and energetic measures be adopted, the attack may be staved off.

*Gangrene.*—At first there is excessive effusion into the scrotum, penis and adjacent parts, which is not reduced by puncturing, but appears to become solidified as soon as thrown out. No healthy suppuration takes place in the original wounds, but an offensive ichorous discharge sets up, the effusion continues, advances forward to the sternum and up the perineum, the parts becoming cold and insensitive. The animal dies a lingering death from exhaustion.

*Hœmorrhage.*—Externally this is slight and easily controlled by cold and pressure. All the deaths reported have been caused by internal hœmorrhage. This is due, I think, either to accidental causes or to the artery having been divided too high up, the divided extremity being drawn within the abdomen. This is less likely to occur in older horses where the testes are more pendulous.

*Tetanus.*—It occurs under all circumstances and is quite uncontrollable. I have seen many cases of Tetanus, but none so acute and rapid in causing death as cases consequent on castration. The disease may occur any time between the 4th and 27th day after operation, but the average is fifteen days.

There is one other surgical complication on which I wish to make a few remarks, and that is Hernia. Of the 10,305 cases there were ten deaths from this cause.

During the struggle of the animal at the time of operation, a portion of the intestines are occasionally forced into the scrotum. In these cases, if it is possible to reduce the hernia quickly, the patient has a good chance of recovery. It is much more serious, however, when the protrusion takes place after the operation when the horse is standing in his stall, as there is then usually a greater length protruded and great fear of injury to the intestine.

A rise of temperature takes place on the third day after the operation, continuing until the 8th and gradually falling until the normal standard is resumed on the 13th day.

In 1856 a series of experiments were conducted by Mr. James Thacker relative to the advantages or disadvantages of the different methods of operating. The ligature, actual cautery, caustic clamps and the scraping method were all tried and the results were in favor of the latter method. Further experiments were carried out by Mr. Shaw and the late Mr. Richardson, V.S., with regard to the relative merits of the scraping method and the actual cautery. These also resulted in favor of the scraping.

